# The influence of income on medical school admissions in Canada: a retrospective cohort study

**DOI:** 10.1186/s12909-020-02126-0

**Published:** 2020-07-01

**Authors:** Tyler Pitre, Alexander Thomas, Kyle Evans, Aaron Jones, Margo Mountjoy, Andrew P. Costa

**Affiliations:** Michael G. DeGroote School of Medicine Waterloo Regional Campus, McMaster University, 10 Victoria Street South, Kitchener, N2G 1C5 Canada

**Keywords:** Admissions, Socioeconomic status, Income, Medical admissions, Undergraduate medicine, MCAT, GPA, MMI, CASPer

## Abstract

**Background:**

The socioeconomic status of applicants to Canadian medical schools has been understudied in the past two decades. Institutional efforts have been made to address the lack of socioeconomic diversity across Canada during this time. We investigated the income characteristics of medical school applicants, as well as the relationship between applicant income and offer of admission, to characterize the current state of socioeconomic diversity in medical admissions.

**Methods:**

We conducted a retrospective cohort study on 26,120 applicants at one Ontario medical school from 2013 to 2018. Characteristics of applicants who were offered admission were compared to the general population and applicants not offered admission. Regression analyses were used to investigate the association between median total neighborhood income and successful admission.

**Results:**

The median total neighborhood income for medical school applicants was $98,816, which was approximately $28,480 higher than the Canadian general population. Those not admitted to the medical school had a median total neighborhood income of $98,304 compared to $105,984 for those offered admission (*p* < 0.001). This trend was seen in every province and territory in Canada. Median total neighborhood income was a predictor of an offer of admission; applicants in the >75th percentile income group had 54% increased odds of being offered admission when compared to applicants in the <25th percentile in our unadjusted model. Income was not significant in our adjusted models but showed that the income medians drastically shifted between pre-interview and post-interview periods, from $98,816 to $104,960 (*p* < 0.001).

**Conclusion:**

Medical school applicants are from higher economic strata compared to the general population. Despite already representing a high economic stratum, a higher median total neighborhood income relative to other applicants was associated with an offer of admission.

## Background

Selecting applicants for admission to medical school is a critical responsibility in medical education. The admissions process to Canadian medical schools is exceedingly competitive. Since most medical students graduate to practice in Canada, Faculties of Medicine must address the ability of future physicians to serve the diverse needs of individuals and communities first through their admissions process.

Concerns have been raised about the dearth of economic diversity among Canadian medical school students and the systemic barriers faced by potential applicants of lower socioeconomic status, such as the high application costs and lack of access to mentors [1]. Decades old research by Dhalla et al. (2002) demonstrated that Canadian medical students had higher average family income, parents with greater formal educational achievements, and were disproportionately from urban areas compared to the general population [2, 3].

In 2010, the Association of Faculties of Medicine of Canada (AFMC) had encouraged Canadian medical schools to support and select a representative mix of medical student candidates, in order to uphold their social mandate and to graduate a physician workforce better able to care and advocate for a diverse population [4]. Diversity in medicine is an important area of study, including wide ranging topics such as cultural competence, linguistic and racial diversity. For example, patient trust is improved by interaction of patients with doctors of similar ethnicity and background [5]. Furthermore, there is evidence that cultural competence training for health care workers leads to better patient outcomes [6]. For our purposes, we address socioeconomic diversity, defined as having students from different income levels and social backgrounds. In our models, we used median neighborhood income as a measure of applicant income, which does not capture individual income but is meant to be a surrogate measure of socioeconomic status.

The AFMC hypothesized that having physicians from a diverse socioeconomic background would be better able to understand unique health challenges and therefore help improve patient care [1]. Yet the socioeconomic status of Canadian medical students has been understudied in Canada, and – to our knowledge – the socioeconomic status of the applicant pool and its effect on receiving an offer of admission has not been studied. Importantly, there has been no studies to update whether there has been improvement in the socioeconomic makeup of medical school classes since the AFMC’s call to action.

Our objective was therefore to describe the socioeconomic status of medical school applicants, those offered admission and whether income is an independent predictor of success using neighborhood income as a proxy measure for socioeconomic status. In addition, we aimed to identify what components of an applicant’s application was associated with an offer of admission.

## Methods

### Study design and data sources

We conducted a retrospective cohort study of applicants to McMaster University’s Michael G. DeGroote School of Medicine from 2013 to 2018. McMaster’s Undergraduate Medical Program (initiated in 1969) is a three-year program that admits approximately 200 students each year. In 2016/17, 74.6% (4974/6672) of all applicants to an Ontario medical school applied to McMaster and 21.4% (206/964) of all admitted/registered medical students in Ontario medical school were for McMaster [7].

We obtained applicants’ admission data for application years spanning 2013 to 2018 inclusive, from two separate files, from two different sources. The first source is from the Ontario Medical School Application Service (OMSAS). All Ontario medical schools use this service for applicants to apply, which provides each applicant a unique identification number. The OMSAS data file contained a parental postal code and Ontario medical school application number. The McMaster data file included OMSAS application numbers, application year, and whether applicants were offered admission (vs. not offered/waitlisted) and, if offered, whether the offer was accepted, declined, or deferred. Data files were combined using common Ontario medical school application numbers. We excluded repeat applicants, assuming that applicants would have used the same Ontario medical school applicant number for subsequent applications. We classified any offer of admission as such, regardless if the offer was accepted, declined, or deferred. Also available in the McMaster file were the applicants’ demographic data, including age category, residency status (Ontario versus out-of-province), identified sex, and the postal code of their home address during high school. Admission testing criteria data included applicants’ undergraduate university Grade Point Average (GPA), Medical College Admissions Test (MCAT) Critical Analysis and Reasoning (CARS; formerly Verbal Reasoning) score, Multi-Mini Interview (MMI) mean score, and CASPer scores. MCAT scores range from 118 to 132 with a median score of all test takers of approximately 125 for each section. As McMaster University only uses the CARS score, we only had access to this data point. CASPer z-scores are utilized as these are typically cited in the literature rather than absolute scores [8, 9]. GPA is a standardized score out of 4.0, reflecting undergraduate academic success. McMaster uses a two-part formula to determine who receives an offer of admission. The first part of the formula is a pre-interview score, which is a composite of 33% for each of the MCAT, GPA and CASPer scores. The second part of the formula is a post-interview score, which is a composite of 70% for MMI and 15% for GPA and MCAT respectively. Lastly, McMaster also preferentially interviews 80–90% of its applicants from Ontario, making geography an important variable to correct for in our model.

We incorporated a proxy measure of applicants’ neighborhood income by including the 2015 median total household income for each applicants’ home postal code during high school. Specifically, using the publicly available Postal Code Conversion File (PCCF), we matched each applicants’ postal code to the most appropriate dissemination area (DA, a relatively stable geographic unit comprised of 400–700 individuals) from the 2016 Canadian Census in order to capture the 2015 median total neighborhood income. This method is a well-established mechanism of estimating neighborhood income [2, 10–12]. However, using a DA as a comparative measure of median total household income for individual applicants may drastically underestimate or overestimate individuals within the DA. Therefore, the results need to be viewed within this context. An applicant’s postal code of their home address during high school was considered ideal given that it defines the family household neighborhood area during a critical scholastic and developmental life stage.

### Statistical analysis

Applicants’ demographic data and admission testing scores were compared between those who were and were not offered admission. In addition, a sensitivity analysis was completed to rule out potential confounders by running the analysis without Ontario applicants included. This was done to protect from concentrated applicants in neighborhoods in the Greater Toronto Area with disproportionately higher neighborhood incomes, as too ensure generalizability. Binary logistic regression was used to characterize the relationship between an offer of admission and applicants’ median neighborhood income. The neighborhood income variable was transformed into different variables representing quartiles of neighborhood income for comparison. Age, sex, application year, and residency (Ontario vs out-of-province) were included in the analysis to control for potential confounding and modifying effects that could also influence an offer of admission.

A second binary logistic regression was performed, first correcting for GPA, MCAT, CASPer and then MMI, GPA and MCAT, to investigate whether the results remained significant. These were modelled to reflect McMaster’s pre and post interview formulas. The first model included all individuals applying, whereas the second model included only those applicants who had an MMI score, which compromised 2973 applicants total. These models only include year’s 2016 to 2018 inclusive as only the newer MCAT CARS scores were used, although sensitivity analysis using the older verbal reasoning (VR) scores yielded similar results. However, because McMaster’s formula for pre and post interview scores are determined solely by these factors, we hypothesize neighborhood income would play little to no role, as there would be no discretion for neighborhood income in this model.

Analysis was done to rule out multicollinearity between variables used. We also performed bivariate Pearson coefficient analysis on multiple admissions scores, offer of interview and neighborhood income to determine which elements were most highly associated with an offer of admission. All data analysis was completed using the Statistical Package for the Social Sciences (SPSS) software, version 24 for Mac (IBM, Armonk, NY).

### Ethics approval

Given the use of secondary de-identified data, strict information security, and institutional sponsorship/oversight, this project was granted a waiver of full review by the Hamilton Integrated Research Ethics Board based on low risk / quality improvement. Administrative review and release were granted by the admissions committee and assistant dean of medicine.

## Results

**Fig. 1:**
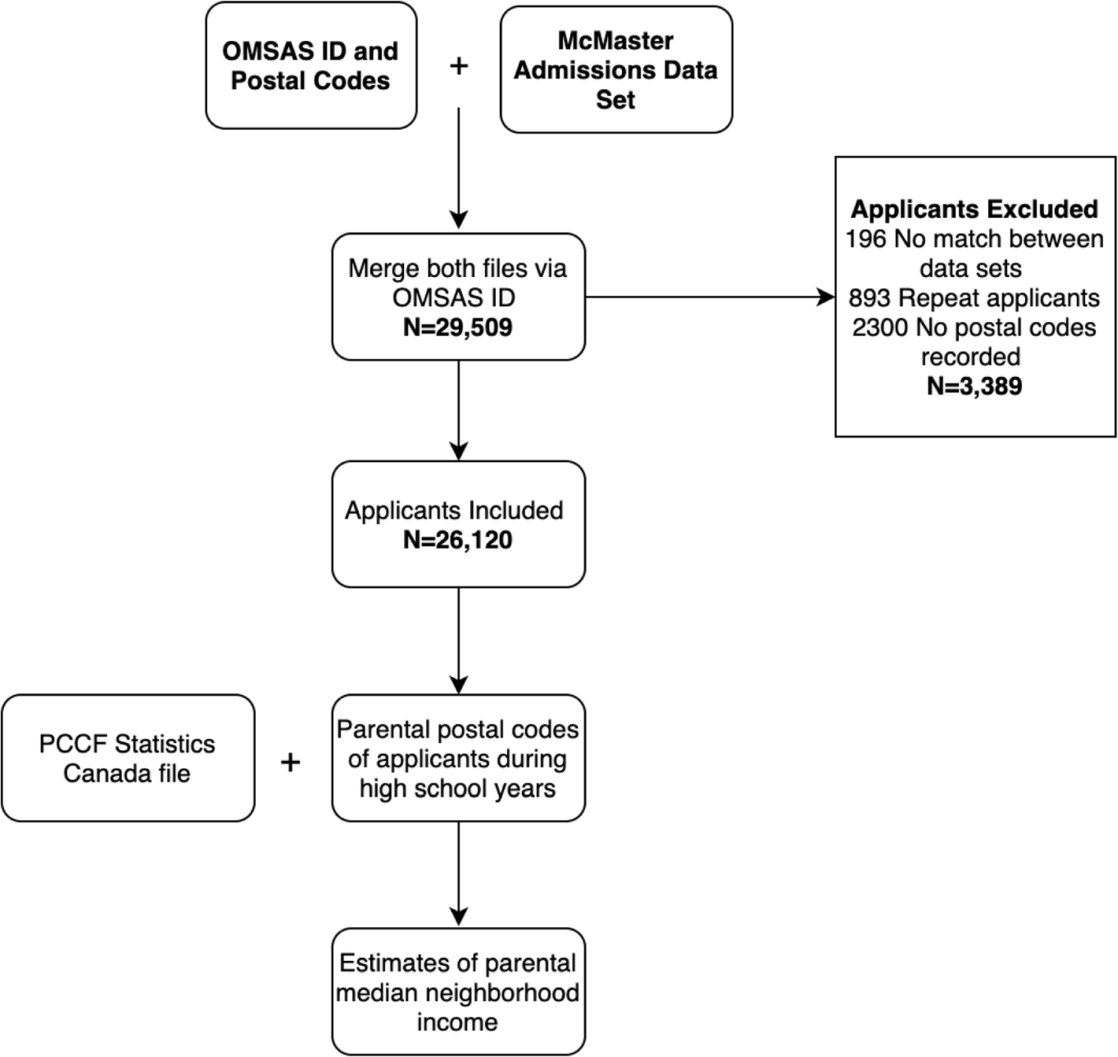
Flow chart summarizing the study process of file merger, data inclusion and exclusion methods

We received data on 29,509 applications. Two separate data files were received, one from McMaster and the other from OMSAS. These files were merged using Ontario medical school application numbers, which was the common variable between the data sets. One hundred and ninety-six applicants were dropped during the merge because there were no matches between data sets, and an additional 3193 applications were dropped because a postal code was not recorded for these applications or repeat applications were identified, and therefore could not be included in the analysis. We were left with 26,120 applications where we could determine a median neighborhood income. Figure 1 is a flow chart representing the process outlined above.

### Demographics of socioeconomic diversity

**Fig. 2:**
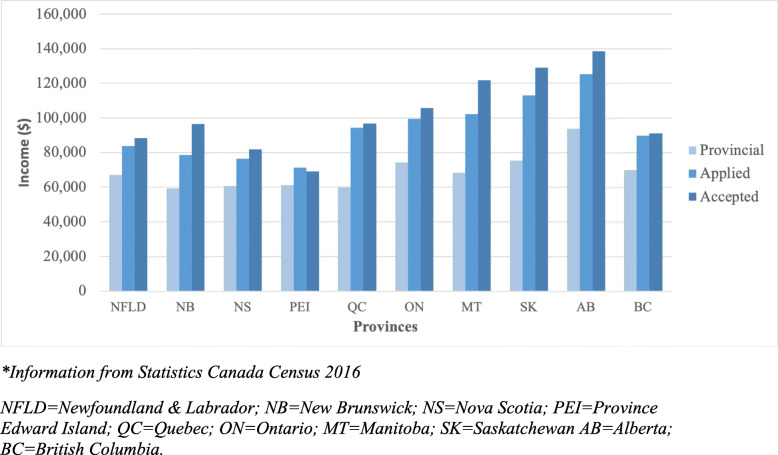
Income distribution of applicants by province for all and accepted applicants, compared against their respective provincial total median household incomes*. *Information from Statistics Canada Census 2016. NFLD = Newfoundland & Labrador; NB=New Brunswick; NS=Nova Scotia; PEI=Province Edward Island; QC = Quebec; ON=Ontario; MT = Manitoba; SK=Saskatchewan AB = Alberta; BC=British Columbia

**Fig. 3:**
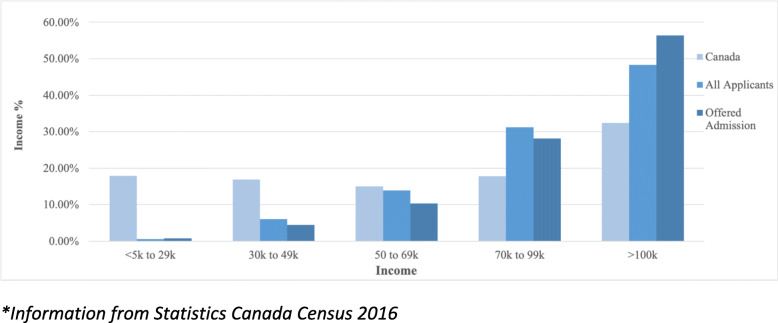
Income distribution of all Applicants and Applicants offered admission, compared to Canadian population*. *Information from Statistics Canada Census 2016

The mean age at the time of application was 23 years old. The female to male ratio was 14,397 (55.1%) to 11,793 (44.9%). In addition, 19,628 (75.0%) of the applicants came from Ontario, whereas 15% came from outside of Ontario, representing every province and territory. The total median neighborhood income of those applying was $98,816. Applicants who were offered admission had a statistically higher median neighborhood income of $105,984.00 (*p* = < 0.001). Figure 2 compares the provincial median incomes with all, and accepted applicant’s median neighborhood incomes and Fig. 3 compares the median total household income distribution of Canadians, all and accepted applicants’ neighborhood income. These results are in keeping with a 2012 survey study that examines the economic diversity of medical school students [11]. Table 1 summarizes the results of the descriptive statistics.

**Table 1:** Characteristics of medical school applicants by offer of admission, among applicants at McMaster University (2013 to 2018, *N* = 26,120)

**Characteristic**	**All Applied (*N*** **= 26,120; 100%)**	**Offered Admission (*N*** **= 1777; 6.8%)**	**Not Offered (*N*** **= 24,343; 93.2%)**	***p*-value**
**Age (Mean, SD)**	23.1, ±3.2	22.0, ±2.6	23.2, ±3.2	< 0.001
**Sex (%, n)**				< 0.001
Female	55.1%,14,397	57.8%, 1026	54.9%, 13,412	
Male	44.9%, 11,723	42.2%, 750	45.1%, 11,021	
**Geography (n, %)**				< 0.001
Ontario	19,628, 75.0%	1547, 87.0%	18,081, 74.3%	
Quebec	591, 2.3%	11, 0.6%	580, 2.4%	
Atlantic	1016, 3.9%	36, 2.0%	980, 4.0%	
Prairies	2240, 8.6%	86, 4.8%	2154, 8.9%	
British Columbia	2614, 10.0%	97, 5.4%	2517, 10.3%	
Territories	31, 0.1%	0, 0%	31, 0.1%	
**Income**				
Median Income $ (n, IQR)	98,816 (123,392-74,752)	105,984.00 (130,816-81,770.50)	98,304.00 (123,392-74,752)	< 0.001
≤ 69 k (%, n)	20.5%, 5365	15.5%, 275	20.0%, 4887	
70 k–99 k (%, n)	31.2%, 8136	28.1%, 500	31.2%, 7623	
≥ 100 k (%, n)	48.3%, 12,619	56.4%, 1002	48.8%, 11,923	
**Income Excluding Ontario** $ (n, IQR)	97,024.00 (123,520-73,131)	109,952, (132,608-78,496)	96,576, (123,136 -72,533)	< 0.001
**MCAT (Mean, SD)**				
Verbal Reasoning	9.4, ±1.8	11.1, ±1.1	9.2, ±1.7	< 0.001
CARS	126.2, ±2.3	129.2, ±1.5	126.0, ±2.3	< 0.001
**Grade Point Average (Mean, SD)**	3.6, ±0.4	3.9, ±0.1	3.6, ±0.4	< 0.001
**CASPer (Mean, SD)**	4.6, ±1.2	5.7, ±0.9	4.5, ±1.2	< 0.001
**MMI (Mean, SD)**^a^	6.4, ±1.1	7.0, ±0.7	5.4, ±0.8	0.10

Abbreviations: *SD = Standard Deviation, IQR = Interquartile Range, Household income = 2015 total income of households for most appropriate dissemination area.; MCAT = Medical college admissions test. CARS=Critical Analysis and Reasoning section. MMI = Multi-Mini Interview, Household income = 2015 total income of households for most appropriate dissemination area*

^a^Only 2973 have an MMI score, as they represent the cohort of applicants offered an interview after the pre-test score

Our sensitivity analysis revealed consistent results; when excluding Ontario applicants, the total median neighborhood income was approximately $97,024.00 for all applicants and $109,952.00 for accepted applicants (*p* = < 0.001).

### Results of regression analyses

There was a statistically significant relationship between applicants’ median total neighborhood income and an offer of admission in our unadjusted model. When comparing quartiles of neighborhood income, there is an increase in odds of admission offer with each increase in neighborhood income quartile. In comparison with an applicant in the <25th percentile to an applicant in the >75th percentile, there is an increase in odds of 54% for an offer of admission. The Naegelkerke’s R^2^ result for this model was 0.06. Table 2 summarizes the results of this model.

**Table 2:** ^a^ Adjusted associations between demographic characteristics and Offer of Admission, among Medical School Applicants (2013 to 2018, *N* = 26,120)

**Variable**	**Odds Ratio (Adjusted)**	***p*-value**	**95% CI**
**Neighborhood Income**			
< 25th percentile	1	< 0.001	
25th to 49th percentile	1.2	0.005	1.06 to 1.44
50th to 75th percentile	1.4	< 0.001	1.21 to 1.62
> 75th percentile	1.5	< 0.001	1.34 to 1.78
**Age**			
Per increase in 1 year	0.8	0.019	0.83 to 0.87
**Sex**			
Female	1		
Male	0.9	0.02	0.81 to 0.98
**Residence statu**^b^			
Out of province	1		
Ontario	3.0	< 0.001	2.56 to 3.63

^a^This model includes all applicants applying, with *N* = 26,120

^b^Residence status is used by our university to determine offer of interview

The binary logistic regression models, adjusted for GPA, MMI, CASPer and MCAT scores, did not show statistically significant results with regard to neighborhood income. In the first model, there was a nonsignificant trend that showed as your income increases, your odds of being offered admission increases. In the second model, there is a contradictory trend. Importantly, the neighborhood income of the applicants in the first model was $98,816 and the neighborhood income of the applicants of the second model is much higher at $104,960 (*p* < 0.001). Importantly, the models need to be interpreted with the understanding the variables in the model are completely determined by the outcome, therefore there is little discretion for other variables to have an impact. The Naegelkerke’s R^2^ result for these models were 0.7 and 0.8 respectively. Tables 3 and 4 summarize these results.

**Table 3:** ^a^ Second model: GPA, MCAT, and CASPer (Pre-interview score)

**Variable**	**Odds Ratio (Adjusted)**	***p*-value**	**95% CI**
**Income**			
< 25th percentile	1	0.866	
25th to 49th percentile	1.1	0.692	0.76 to 1.51
50th to 75th percentile	1.0	0.810	0.75 to 1.46
> 75th percentile	1.1	0.426	0.82 to 1.60
**Admission Tests**			
MCAT	2.9	< 0.001	2.68 to 3.15
GPA	2.3	< 0.001	2.0 to 2.4
CASPer	9.8	< 0.001	8.1 to 11.8
**Age**			
Per increase in 1 year	1.0	0.432	0.97 to 1.1
**Sex**			
Male	1	< 0.001	
Female	1.5	< 0.001	1.23 to 1.94
**Residency status**^b^			
Out of Province	1	0.998	
Ontario	4.3	0.275	0.31 to 58.3

^a^This represents the first part of McMaster’s formula for selection of applicants, representing 26,120 applicants

^b^Residence status is used by McMaster university to help determine an offer of interview

**Table 4:** ^a^ Third model: GPA, MCAT, and MMI (Post-interview score)

**Variable**	**Odds Ratio (Adjusted)**	***p*-value**	**95% CI**
**Income**			
< 25th percentile	1	0.885	
25th to 49th percentile	0.8	0.434	0.42 to 1.45
50th to 75th percentile	0.9	0.700	0.48 to 1.63
> 75th percentile	0.8	0.579	0.46 to 1.55
**Admission Tests**			
MCAT	1.7	< 0.001	1.43 to 1.92
GPA	1.5	0.001	1.20 to 1.70
MMI	51.2	< 0.001	31.9 to 82.0
**Age**			
Per increase in 1 year	1.00	0.998	0.89 to 1.1
**Sex**			
Male	1	0.555	
Female	1.1	0.766	0.71 to 1.58
**Residency status**^b^			
Out of Province	1	0.784	
Ontario	1.5	0.248	0.74 to 3.27

^a^This represents the second part of McMaster’s formula for selection of applicants, with 2973 applicants included

^b^Residence status is used by our university to determine offer of interview

### Pearson coefficients analyses

We performed Pearson coefficients for an offer of admission to academic scores, offer of interview and neighborhood income, which showed that an offer of interview correlated the strongest with overall chance of being offered admission, with a positive Pearson coefficient of 0.75. No significant collinearity was found between these variables. Table 5 summarizes the results of the Pearson coefficients.

**Table 5:** Bivariate Pearson coefficient on academic scores, offer of interview and neighborhood income

**Variable**	**Pearson Coefficient**	***P*-value**	
**Admission**	1		
Interview Invite	0.75	< 0.001	
MCAT	0.34	< 0.001	
CASPer	0.26	< 0.001	
GPA	0.18	< 0.001	
Income	0.05	< 0.001	

## Discussion

Our study confirmed that applicants from areas with higher total neighborhood incomes were more likely to apply and to be offered admission to medical school. We found that the median neighborhood income of all applicants was 33.7% percent higher than the Canadian median total household income. Applicants that were offered admission had a 40.4% higher neighborhood income than the national median [13]. Furthermore, 48.3% of all applicants had a median total neighborhood income bracket greater than or equal to $100,000. We demonstrated that applicants from greater than or equal to $100,000 form most successful applicants, with 56.4% coming from areas with median neighborhood incomes greater than or equal to $100,000. In addition, we showed that our analysis is not biased by our large Ontario cohort.

### Regression models

Although neighborhood income in our adjusted binary regression model was not significant when correcting for MCAT, CASPer, MMI and GPA, this is to be expected, as McMaster’s pre-interview score is generated as a function 33% for each component and therefore leaves no discretion. Similarly, the MMI is weighted at 70% post-interview score, with MCAT and GPA accounting for 15% each. Interestingly, our first and second model, summarized in Tables 3 and 4, show contradictory results. We believe this to be an important finding. The first model represents the phase of the application process where academic scores such as MCAT, GPA and CASPer completely determine an offer of interview. This group of applicants had a median neighborhood income of $98,816. Interestingly, in the second model, which is a smaller cohort of successful applicants from the first model, had a significantly higher income of $104,960, which is approximately the income of applicants offered admission. This suggest that the applicants of lower socioeconomic background are disadvantaged in the first round of the application process. This is in keeping with an established literature that success in these exams correlates with income [14, 15]. In the second round, the interpretation is less clear. Either income is less of a factor, perhaps from the MMI having so much weight (70% of the total score) or because the applicant cohort is now of significantly higher incomes, and therefore there is no variability for the model to test. Although it has been previously established that medical school applicants tend to be from higher income households [2, 11], our study is significant because, despite institutional calls to action and knowledge of these facts for decades, we show that there is still significant disparity in the socioeconomic diversity of medical school admissions. It also points to a particular aspect of the application process, namely the pre-interview process, that may be disadvantageous to lower socioeconomic applicants.

### Applicant advantage in an already biased cohort

We found that applicants from areas with a greater median neighborhood income tend to be accepted into medical school. Measures used to rank students, such as the MCAT, can have significant financial barriers, with a large financial market for preparation courses. This is in keeping with literature suggesting that MCAT, other admissions tests and application fees correlate significantly with applicant’s income [14–18]. This is also consistent with our discussion above, namely that the part of the application process that is determined by these scores may be influenced by socioeconomic status. Importantly, neighborhood income remains a predictor of success despite the significant skew in all applicants who already are disproportionately from neighborhood incomes of above $100,000 and is demonstrated visually in Fig. 2.

### Applicants from higher SES are more likely to apply

To our knowledge, we are the first to demonstrate that applicants from areas with a greater median neighborhood income are more likely to apply to medical school in the first place. These results remain significant even when dispensing with our large Ontario cohort. These findings suggest that there are critical advantages in higher income areas that result in an over-representation in medical school applicants. Some advantages may directly relate to the measures used in an offer of admission, as they are often associated with expensive preparatory courses that can deter many low-income applicants from applying. Application fees are expensive and have been identified as a potential limiting factor for persons of lower socioeconomic status [19]. Taken together, these factors can aggregate and can form a perception that many potential applicants have regarding their chances of being offered admission. For example, rural students in Newfoundland reported feeling they would have a harder time being accepted to medical school and that medicine was not promoted as a potential career [16]. In addition, high school applicants in Southwestern Ontario reported feeling that finances were a potential barrier to being accepted into medical school. Interestingly, these feelings correlated with parental neighborhood income and circumstance [20]. These examples exemplify self-selection, which are the preconceived notions that act to limit potential applicants from applying in the first place.

### Offer of interview, admission and income

Interestingly, our Pearson coefficient analysis reveals an important correlate with an offer of admission, namely that an offer of an interview correlates strongest with an offer of admission. We found that on an individual basis, an offer of interview correlated more with an offer of admission than any of the academic score or neighborhood income. Interestingly, based on our models in Tables 3 and 4, this also represents a significant change in the neighborhood income distribution of applicants. Therefore, indirectly representing a bias for applicants of lower socioeconomic status. From our knowledge, we are the first report this particular finding.

### Impact

Our study contributes to the broader debate on the importance of medical school diversity and quantifies the magnitude of the current problem. Specifically, we are the first to report on the direct relationship that neighborhood income has in medical admissions as well as characterizing applicants to medical school. First, at our institution, this study provides an in-depth analysis of the current status of our socioeconomic diversity and will direct focus on further study to inform policy changes. Second, this study may galvanize medical programs across Ontario and Canada to characterize their respective economic diversity profiles and continue to address this issue. Importantly, it should galvanize a discussion around the concept of hidden biases. There is no direct measure of socioeconomic status in medical admissions, but the assessments used in determining success in medical admissions have positive correlations with ascending affluence. Although this study is at one institution, our data set includes approximately 74.6% of all Ontario medical school applicants and 21.4% of all admitted registrants to an Ontario medical school [7].

## Limitations

Our study has many strengths but several notable limitations. Although our data set is large, our study includes only a single institution and therefore our findings may not generalize to all medical schools in Canada. However, our study includes relatively large contributions from every province and territory and is larger than any other study on the subject to date. Furthermore, McMaster is recognized as a leader in inclusive medical admissions. They are the developers of the MMI and CASPer, as well as research on equity in medical school [21–25]. In addition, our socioeconomic proxy measure of median neighborhood income is based on postal codes and may not be reflective of the income of all medical applicant households. Furthermore, the neighborhood income of rural households is difficult to estimate in this manner [26]. However, we suspect that this would only make our findings more conservative. Finally, although our report identifies important associations, we cannot determine with certainty the causative mechanism(s) behind our findings, although we have postulated reasonable theories.

## Conclusion

Our analysis demonstrated that the general pool of medical school applicants is skewed towards the upper quartiles of neighborhood income and are not representative of the national income distribution. Furthermore, neighborhood income is associated with success in the medical school admissions process, although with uncertain etiology. This is despite a recent institutional call to action to improve the socioeconomic diversity of medical school admissions. Future studies should focus on investigating the mechanism of the association to inform efforts to mitigate the bias, in order to fulfill the obligation, set out by the AFMC to encourage a more socioeconomically diverse physician workforce.

## Supplementary information

**Additional file 1: Table 1.** Subgroups of applicants in Tables 3 and 4.

## Data Availability

Due to the sensitivity of the data in the study, we cannot make all data available but individual requests can be made and will be given individual assessment. Requests should be made to the corresponding author.

## Abbreviations

AFMC: Association of faculties of medicine of Canada
MCAT: Medical college admissions test
OMSAS: Ontario medical school application service
VR: Verbal reasoning
MMI: Multi-mini interview
GPA: Grade point average
CASPER: Computer-based assessment for sampling personal characteristics
PCCF: Postal code conversion file
DA: Dissemination Area
SPSS: Statistical Package for the Social Sciences
