# Supplementary material for: The influence of income on medical school admissions in Canada: a retrospective cohort study
Source: BMC Med Educ. 2020 Jul 1;20:209. doi: 10.1186/s12909-020-02126-0 (PMC7329437; doi:10.1186/s12909-020-02126-0)
Supplement: Supplementary file 1 — Additional file 1: Table 1. Subgroups of applicants in Tables 3 and 4. [file 12909_2020_2126_MOESM1_ESM.docx]

**Supplementary Table 1**. Comparative statistics from binary logistic regression seen in tables 3 and 4.

| **Variable (N, SD)** | **Model A** | **Model B** |
| --- | --- | --- |
| Total Number | 26,120 | 2,973 |
| Income ($) | 98,816 (123,392 – 74,752) | 104,960 (129,728 – 82,272) |
| Sex (F/M) | 14397/11723 | 1622/1351 |
| Age | 23.1 +-3.21 | 21 +-2.4 |
| CASPer | 4.58 +-1.2 | 5.7 +- 0.9 |
| MMI* |  | 6.4 +-1.1 |
| GPA | 3.61 +-0.37 | 3.9 +- 0.1 |
| MCAT CARS | 126.0 +-2.34 | 129.0 +-1.5 |

*Only applicants in Model B have an MMI score.
